# Exploring the efficacy of repeated low-level red-light therapy in retarding childhood myopia progression: updated systematic review and meta-analysis

**DOI:** 10.3389/fmed.2026.1713885

**Published:** 2026-01-22

**Authors:** Chen Liu, Yating Zhou, Zongyue Zhan, Xiaofeng Li

**Affiliations:** 1 Department of Ophthalmology, Affiliated Xinhua Hospital of Dalian University, Dalian, Liaoning, China; 2 Department of Ophthalmology, Kunshan Hospital of Traditional Chinese Medicine, Suzhou, Jiangsu, China; 3 Department of Ophthalmology, Shanghai University Affiliated Peace Eye Hospital, Shanghai, China

**Keywords:** myopia, repeated low-level red-light, meta-analysis, systematic review, children

## Abstract

**Background:**

Due to the increasing prevalence of childhood myopia, low-level red-light therapy has surfaced as a non-pharmacological method to mitigate its advancement. This systematic review and meta-analysis evaluates the efficacy of repeated low-level red-light (RLRL) in pediatric populations, providing evidence-based support for its therapeutic usage.

**Methods:**

A search was performed in PubMed, Web of Science, Cochrane Library, Embase, and prominent Chinese databases, including CNKI, VIP, and Wanfang, from their inception until May 13, 2025. This meta-analysis includes randomized controlled trials (RCTs) that matched the inclusion criteria and was analyzed using Stata software, version 18.0.

**Results:**

This meta-analysis included 1,053 participants: 515 in the RLRL and 538 in the control groups. The SMD for SER reduction was 0.84 (95% CI: 0.50, 1.19), SMD for AL reduction was −1.01, (95% CI: −1.60, −0.41), and SMD for SFChT rise was 0.81 (95% CI: 0.65–0.98). There is no major publishing bias.

**Conclusion:**

The meta-analysis found that children with myopia who got RLRL therapy in addition to normal treatment had significantly slower disease progression than the control group.

**Systematic review registration:**

https://www.crd.york.ac.uk/PROSPERO/view/CRD420251034037, PROSPERO 2025 CRD420251034037.

## 1 Introduction

Myopia, often referred to as nearsightedness, is a prevalent visual impairment that typically develops during infancy or early adulthood. The condition arises when the elongation of the eye becomes excessive, causing light rays to converge in front of the retina instead of directly upon it. This optical malfunction contributes to a disconcerting inability to view distant objects clearly. As one of the most prevalent ocular conditions worldwide, myopia affects millions across the globe (1–3). There are 1.41 billion people currently suffering from myopia worldwide, making up nearly 23% of the global population. Estimates suggest that by 2050, the global population of people with myopia will reach 4.76 billion, representing 49.8% of the world’s total (4). In parts of East and Southeast Asia, myopia has become a significant public health concern due to its sharp rise in recent decades (affecting 80–90% of graduating students) (5). Myopia not only affects children and adolescents’ study and life, but may also pose a risk for irreversible and vision-threatening diseases such as retinal detachment, pathologic myopia, and glaucoma (6, 7). Consequently, it is of utmost significance to avert the onset, postpone the advancement of myopia, and mitigate the associated complications arising from axial elongation. Currently, the primary intervention strategies include prescribed multifocal spectacle lenses, low-concentration atropine eye drops, different types of contact lenses such as corneal orthokeratology (OK) with lenses (8, 9). However, all these interventions are associated with their own risks. For example, multifocal lenses are less effective, while OK lenses can cause keratitis and other complications. Furthermore, long-term use of low-dose atropine can result in a variety of ocular symptoms, including increased intraocular pressure and systemic adverse effects. Recently, the prevention and control of myopia through red light irradiation has garnered significant attention, owing to its remarkable efficacy in decelerating the progression of myopia without any adverse effects (10, 11).

In recent years, low-level red-light therapy, functioning within the 600–700 nm wavelength spectrum of visible light (12), has gained significant attention globally as a treatment modality in ophthalmology. This novel approach improves cellular energy production and metabolic activity, thus accelerating tissue repair and regeneration (13). It significantly slows the progression of myopia, protects retinal cells in glaucoma, reduces inflammation associated with age-related macular degeneration, and relieves symptoms of dry eye illness (14). Recent study suggests that consistent use of recurrent low-level red-light (RLRL) therapy may substantially decelerate myopia progression and potentially elicit a hyperopic shift. Experimentation on animals has shown that red light can restrain eye development in primates. After intervention with red light in primates, the growth rates of myopic refraction and axial length significantly slowed down, demonstrating a certain effect of myopia control (15, 16). Based on the finding that red light can inhibit the development of myopia in primates, some scholars have conducted clinical studies on myopia control using red light and have preliminarily explored the impact of red light on myopia control, as a potential new therapeutic for myopia. However, controversies still exist regarding the effectiveness of red-light use. Paradoxically, in experiments involving human subjects, short-term (1 h) exposure to monochromatic red (623 nm) lighting leads to an increase in axial length and choroidal thinning (17). Furthermore, recent international study has commenced investigating the efficacy of repeated low-level red-light therapy in decelerating the advancement of myopia. Hence, this study is a systematic review and meta-analysis to evaluate the effectiveness of RLRL for controlling the myopia.

## 2 Methods

This analysis rigorously complied with the Preferred Reporting Items for Systematic Reviews and Meta-Analyses (PRISMA) standards (18) and is officially registered on PROSPERO (CRD420251034037). Since it is a systematic review, there is no requirement for ethical clearance.

### 2.1 Literature search strategy

A comprehensive literature search was founded in PubMed, Cochrane Library, Embase, Web of Science, CNKI, VIP and Wanfang databases from their inception through May 13th, 2025, and search strategy used MeSH terms and keywords: ‘Myopias,’ ‘Repeated low-level red-light,’ and ‘Randomized controlled trial’. To ensure comprehensive literature coverage, additional searches of the World Health Organization International Clinical Trials Registry Platform (WHOICTRP) and ClinicalTrials.gov for pertinent papers. Additionally, a thorough examination of preprint repositories such as medRxiv and Research Square was conducted to find unpublished research (These platforms provide early versions of the latest research in the biomedical and medical fields). The specific PubMed search methodology is detailed in Table 1, while the remaining databases followed an identical search protocol. Two authors conducted literature research independently and verified the included studies with each other. If there are any disagreements, a third author is consulted to resolve them.

**Table 1:** Search strategy of PubMed.

Search number	Query	Results
#1	“Myopia” [Mesh]	23,258
#2	(myopias[Title/Abstract]) OR (nearsightedness[Title/Abstract])	266
#3	#1 OR #2	23,298
#4	“Red Light” [Mesh]	121
#5	(red-light[Title/Abstract]) OR (light red[Title/Abstract])	10,561
#6	#4 OR #5	10,570
#7	((((((((“Low-Level Light Therapy”[Mesh])) OR (Light Therapies, Low-Level[Title/Abstract])) OR (Light Therapy, Low-Level[Title/Abstract])) OR (Low-Level Light Therapies[Title/Abstract])) OR (Low Level Light Therapy[Title/Abstract])) OR (Therapies, Low-Level Light[Title/Abstract])) OR (Therapy, Low-Level Light[Title/Abstract])) OR (LLLT[Title/Abstract])	9,223
#8	#6 OR #7	19,542
#9	#3 AND #8	93
#10	randomized controlled trial[Publication Type] OR randomized[Title/Abstract] OR placebo[Title/Abstract]	1,122,869
#11	#9 AND #10	40

### 2.2 Inclusion and exclusion criteria

Studies were chosen according to these criteria: (1) participants under 18 years with diagnosed myopia; (2) inclusion of at least one treatment group utilizing red light therapy; (3) reporting of outcomes such as spherical equivalent refraction (SER), axial length (AL), or subfoveal choroid thickness (SFChT); (4) study design restricted to randomized controlled trials (RCT); and (5) a minimum follow-up period of 3 months. The studies were excluded based on the following criteria: (1) Individuals with additional eye-related conditions in either eye, such as strabismus, binocular vision disorders, or systemic illnesses; (2) non-empirical research, such as literature reviews, case studies, animal trials, or studies without randomized controls; and (3) investigations with incomplete datasets or duplicate publications.

### 2.3 Study selection

All selected studies were cataloged with EndNote 21. Initially, any duplicate entries were weeded out. Following this, two researchers, working independently, screened the studies. Weeding out the studies that did not align with our criteria, based on titles and abstracts. A full-text review was conducted to assess eligibility for the selected articles. Any differences were resolved by consulting with the third researcher.

### 2.4 Data collection and extraction

Both reviewers extracted the following information from the included studies independently: (1) study characteristics: first author, publication year, study design, sample size, and follow-up duration; (2) participant information: age, baseline SER, AL, and SFChT; (3) intervention details: red-light wavelength, output power, and irradiation protocol; (4) outcomes: primary and secondary outcomes. In the absence of clear data in the published study, the research findings were obtained from the original dataset. Due to the varying follow-up cycles of each trial, baseline data before to the low-level red-light intervention and endpoint data for SER, AL, and SFChT post-intervention were extracted, and the differences were computed for analysis. If the original text has provided SER, AL, and SFChT, the change difference directly extracts the data and is used for the final meta analysis. If the data provided in the article is the mean ± standard deviation (SD), it will be recorded directly. When presenting data in the form of 95% confidence intervals (CI), one must use the appropriate formula to calculate the SD before proceeding with the analysis. If the data in the article is the median and quartile spacing, the data should be converted into the mean ± SD before analyzing, data conversion was performed referring to the Cochrane Handbook: calculate interquartile range as IQR = Q3–Q1, estimate SD using the formula SD ≈ IQR/1.35, and approximate mean with median (Mean ≈ Median), assuming the data were approximately normally or symmetrically distributed (19).

### 2.5 Risk of bias assessments

Two blinded independent researchers employed the Cochrane Risk of Bias Tool (ROB) 2.0 (20) to scrutinize the quality of included studies. The ROB 2.0 program evaluates five essential domains of potential bias: randomization, variations from intended interventions, missing outcome data, outcome measurement, and selective result reporting. When discrepancies occurred, a third researcher was involved to facilitate consensus. Two independent researchers estimated the certainty of outcomes using the Grading of Recommendations, Assessment, Development and Evaluations (GRADE) approach (21). This process began by determining the initial certainty level based on study types: RCTs were assigned a high certainty rating, while observational studies were categorized as having low certainty. Subsequently, the certainty of evidence would then be diminished if factors such as risk of bias, inconsistency, or publication bias were evident. Ultimately, the classification of evidence certainty was delineated into four distinct levels: high, moderate, low, and very low. These assessment procedures were carried out using GRADEpro software.

### 2.6 Statistical analysis

The meta-analysis was conducted utilizing Stata 18.0 software. For continuous outcomes, the mean difference (MD) and the 95% CI were chosen for analysis. Heterogeneity was assessed using the *I^2^* statistic and *p-*values (22). If the heterogeneity was low or negligible, a fixed-effects model was applied; otherwise, a random-effects model was employed. Sensitivity analysis was conducted to identify potential sources of heterogeneity. Additionally, Publication bias was analyzed using funnel plots and Egger’s regression test.

## 3 Results

### 3.1 Summary of included research

**Figure 1:**
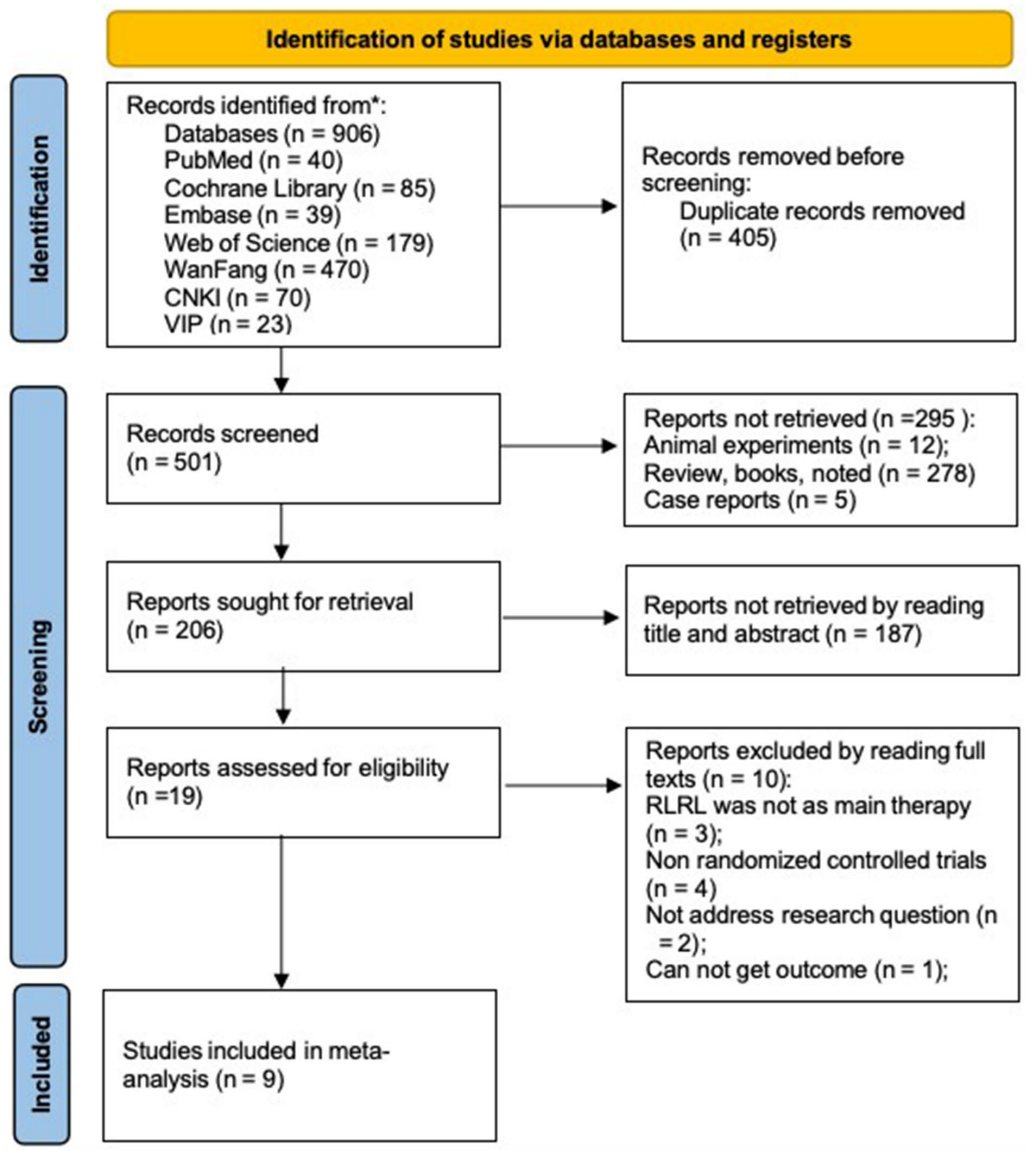
Flow diagram of the selection process.

906 articles were identified from the database, and nine RCTs were selected for this meta-analysis. 1,053 children were included (515 in the experimental group and 538 in the control group). The flow diagram of the study selections shown in Figure 1. Their basic characteristics are shown in Table 2. Among the studies considered, a total of 1,053 individuals were involved. Nine articles provided baseline and endpoint data or the variations in changes of SER and AL, whereas five research recorded the alterations in SFChT. Chinese literature was of inferior quality after assessment and, therefore, was not included in the analysis of this study.

**Table 2:** Characteristics of the studies included in the meta-analysis.

Study	Design	Age	Wavelength of red light (nm)	Irradiation scheme	Group (invention/control)	Baseline of AL (mm)	Baseline of SER (D)	Baseline of SFChT (μm)
Jiang et al. (2022) (23)	RCT	8–13	650 ± 10	2 times/d, 3 min/times, 5 d/w, 1-, 3-, 6-, and 12-month follow-up visits	RLRL+SVS/ SVS	24.54 ± 0.67/24.62 ± 0.86	−2.49 ± 0.92/ −2.67 ± 1.06	NA
Xiong et al. (2024) (24)	RCT	6–14	650	2 times/d, 3 min/times, 5 d/w, 1-,3-,6-month follow-up visits	RLRL+SVS/ SVS	24.38 ± 0.87/24.47 ± 0.58	−2.47 ± 1.39/ −2.22 ± 0.72	251.83 ± 65.27/274.76 ± 63.79
Chen et al. (2022) (25)	RCT	7–15	650 ± 10	2 times/d, 3 min/times, 7 d/w, 1-, 3-, 6-, and 12-month follow-up visits	RLRL+SVS/ LDA	24.48 ± 0.79/24.67 ± 0.98	−2.60 ± 1.17/ −2.59 ± 1.24	NA
Tian et al. (2022) (26)	RCT	6–12	650	2 times/d, 3 min/times, 7 d/w, 6-month	LLRL+SVS/ SVS	24.31 ± 0.92/24.20 ± 0.85	−2(−3.25, −1.25)/ −2(−2.75, −1.25)	290.5 (242, 352.5)/ 296 (244, 352)
Chen et al. (2022) (27)	RCT	6–13	635	2 times/d, 3 min/times, 3-, 6-, 9-, 12-month follow-up visits	LRL + SFS/ SFS	24.62 ± 0.97/24.57 ± 0.76	−2.54 ± 1.04/ −2.29 ± 0.77	259.00 ± 51.46/273.08 ± 54.37
Dong et al. (2023) (28)	RCT	7–12	NA	2 times/d, 3 min/times, 6-month follow-up visits	RLRL+SVS 10%Sham device + SVS	24.7 ± 1.04/24.6 ± 0.96	−3.13 ± 1.91/ −2.82 ± 1.86	NA
Yang et al. (2025) (29)	RCT	8–10	650	2 times/d, 3 min/times, 6-,12-month follow-up visits	RLRL+SVS/ SVS	24.30 ± 0.87 23.93 ± 0.66	−1.13(−1.38 to −1.00)/ −1.13(−1.25 to −1.00)	NA
Xiong et al. (2021) (30)	RCT	7–15	650 ± 5	2 times/d, 3 min/times, 7 d/w, 1-, 3-, and 6-month follow-up visits	LLLT+SVS/ SVS	25.07 ± 0.87/25.07 ± 0.87	−3.39 ± 2.17/ −3.32 ± 1.36	288.61 ± 59.59/286.81 ± 63.67
Deen et al. (2025) (31)	RCT	8–13	650 ± 10 nm	2 times/d, 3 min/times, 5 d/w, 1-, 3-, 6-, and 12-month follow-up visits	RLRL+SVS/ SVS	24.24 ± 0.69/24.54 ± 0.76	−2.26 ± 0.89 −2.16 ± 1.04	NA

NA, not available; RCT, randomized clinical trial; AL, axial length; SER, spherical equivalent refraction; SFChT, subfoveal choroidal thickness; SVS, single-vision spectacle; SFS, single-focus spectacles; RLRL, repeated low-intensity red light; LLRL, low-level red light; LRL, low-intensity red-laser; LLLT, low-level laser therapy; Low-Dose Atropine (LDA).

### 3.2 Quality assessment and bias risk

**Figure 2:**
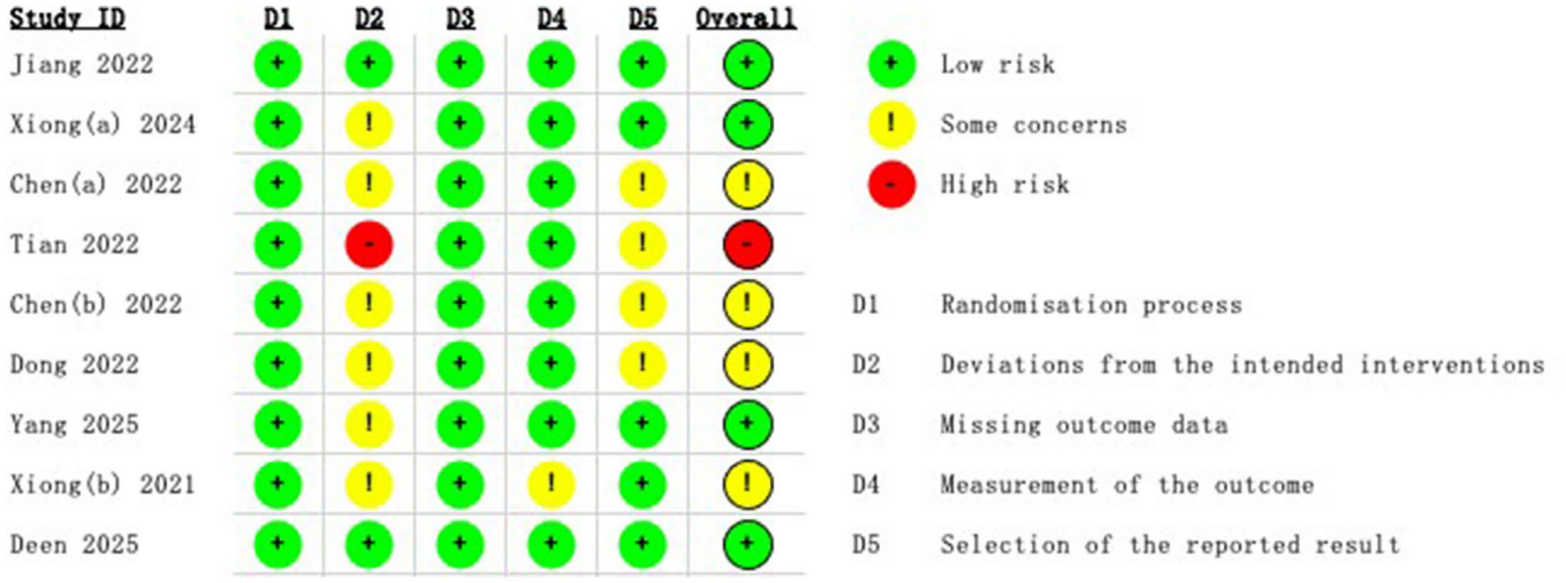
Traffic light plot of the risk of bias assessment of RCTs using ROB2.0.

**Figure 3:**
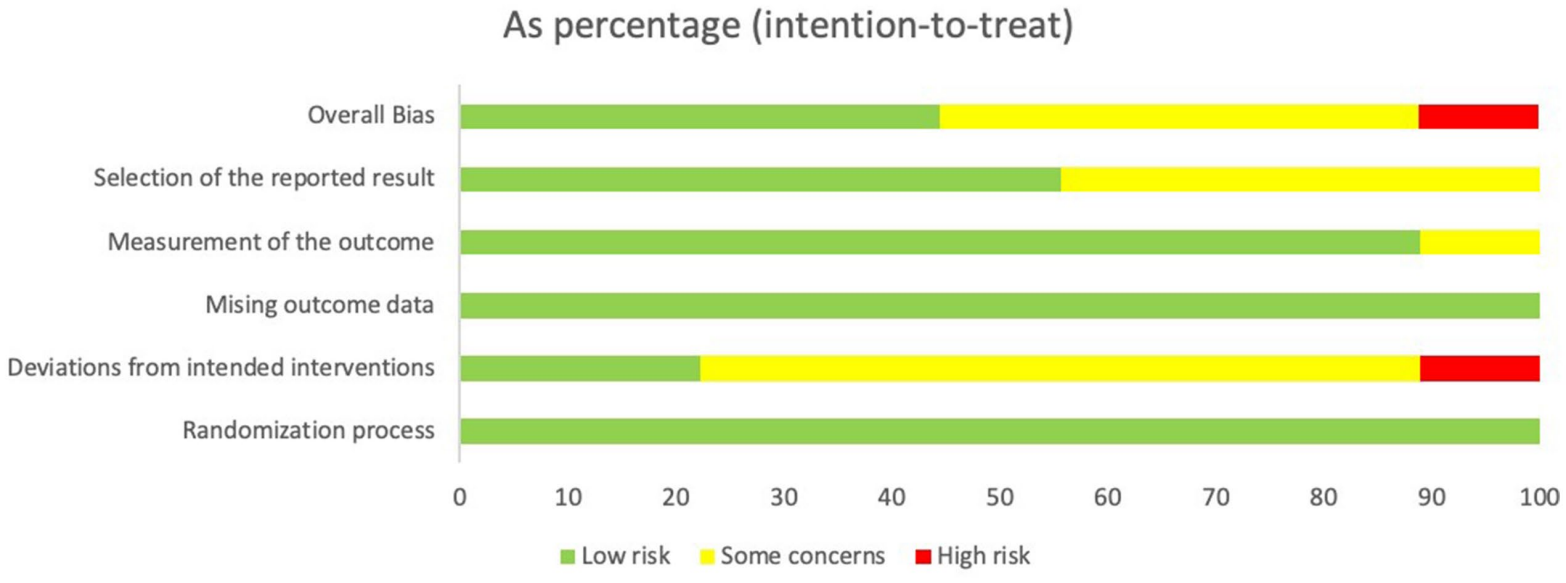
Summary plot of the risk of bias assessment of RCTs using ROB2.0.

The studies were assessed using the ROB 2.0 framework, which analyzes five categories of randomized controlled trials on the risk of bias, in addition to an evaluation of the overall bias present. According to the results, almost all included studies were assessed as having low, moderate, or high risk. The assessment process is shown in Figures 2, 3.

### 3.3 Outcomes subject to meta-analysis

#### 3.3.1 Primary outcome

**Figure 4:**
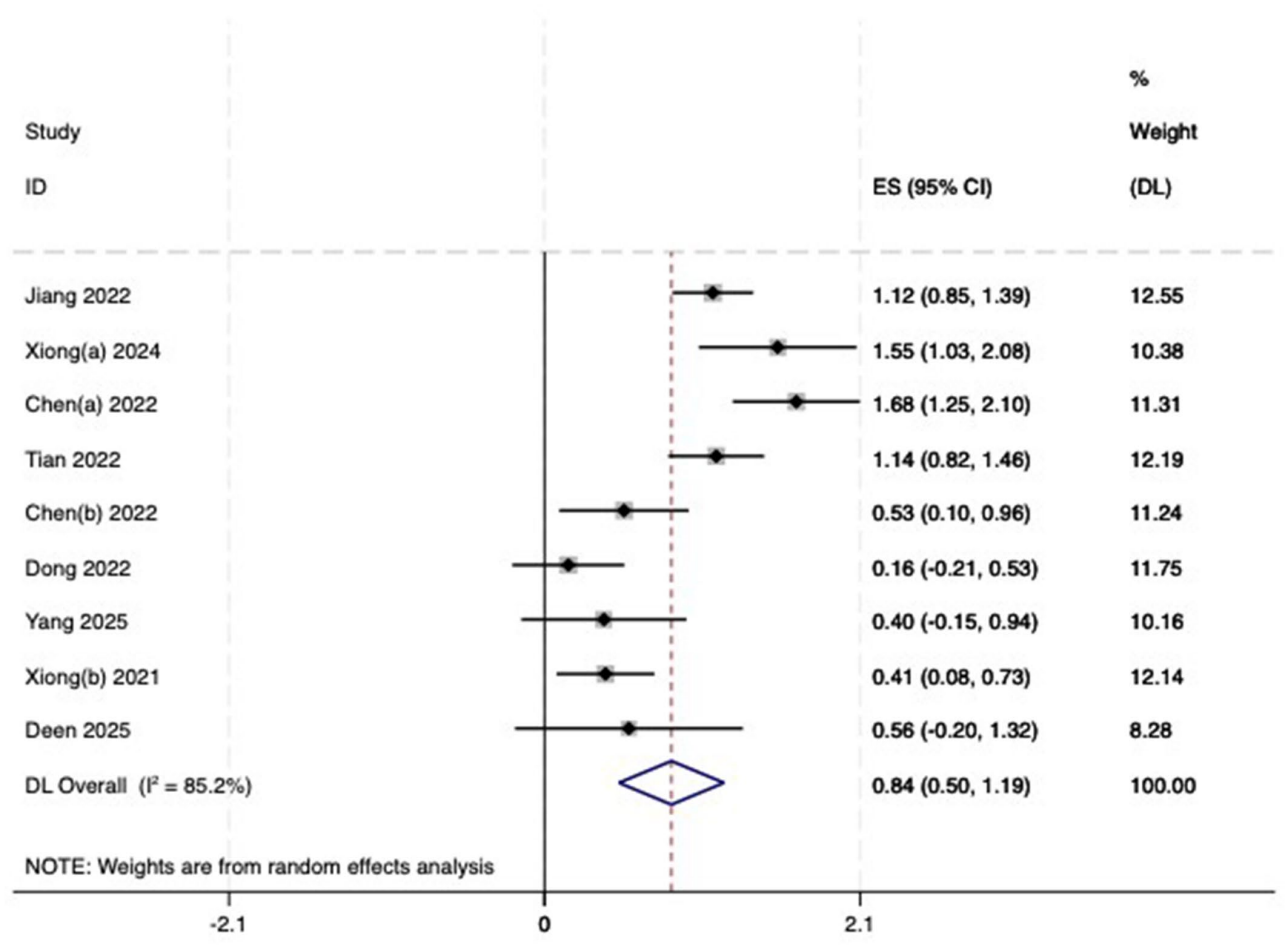
Forest plot of SER.

**Figure 5:**
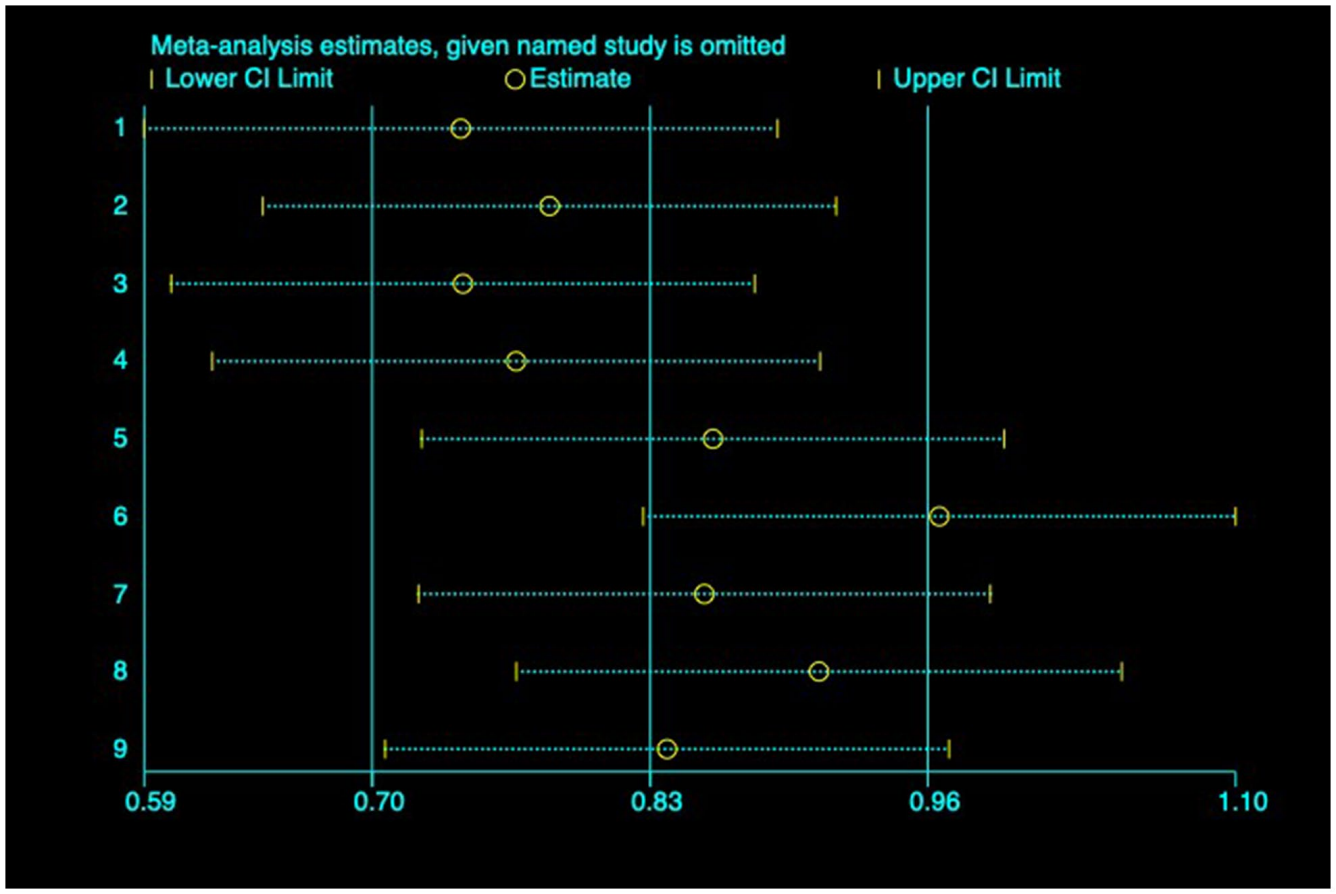
Sensitivity analysis of SER.

**Figure 6:**
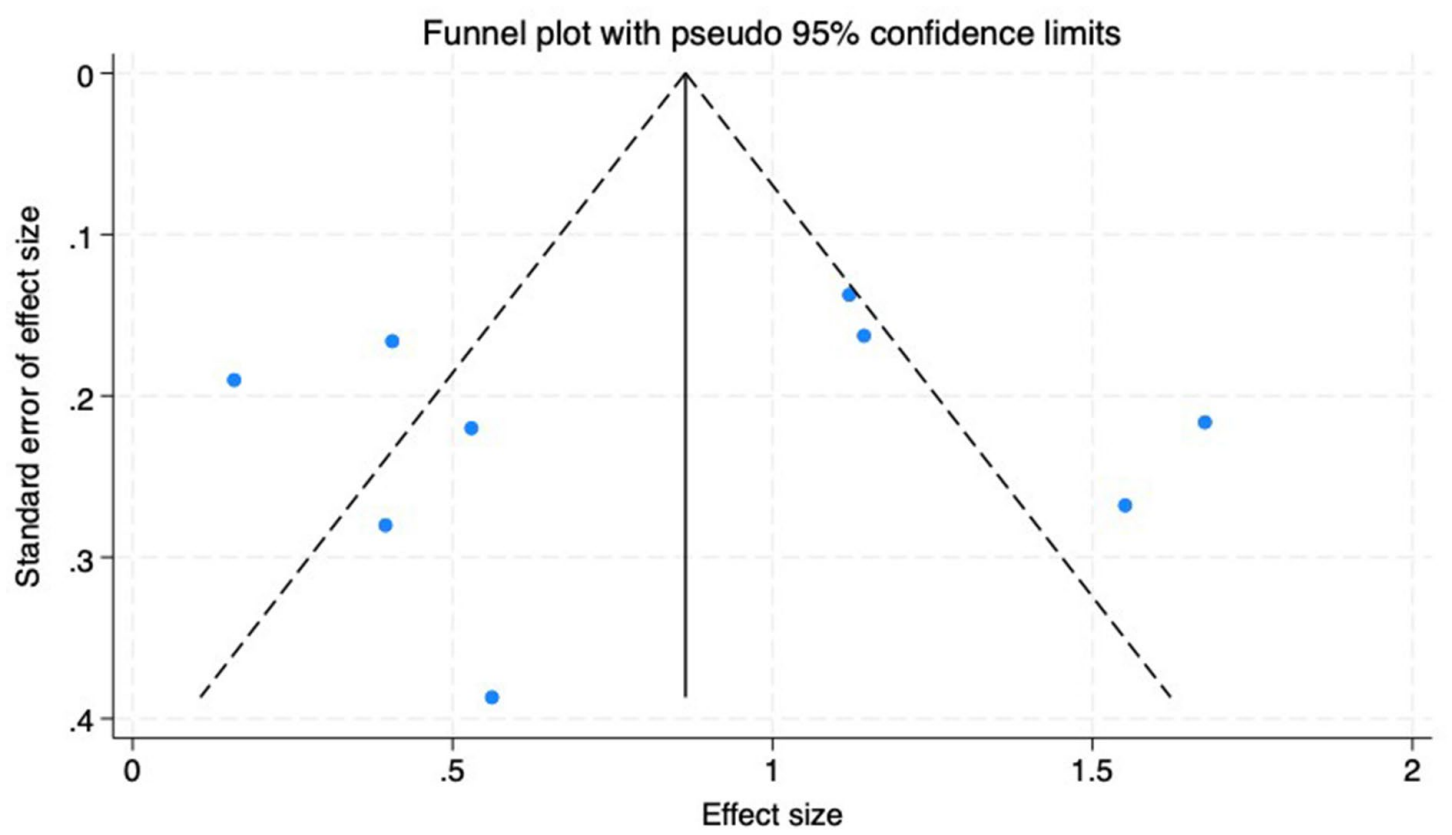
Funnel plot of the SER.

**Figure 7:**
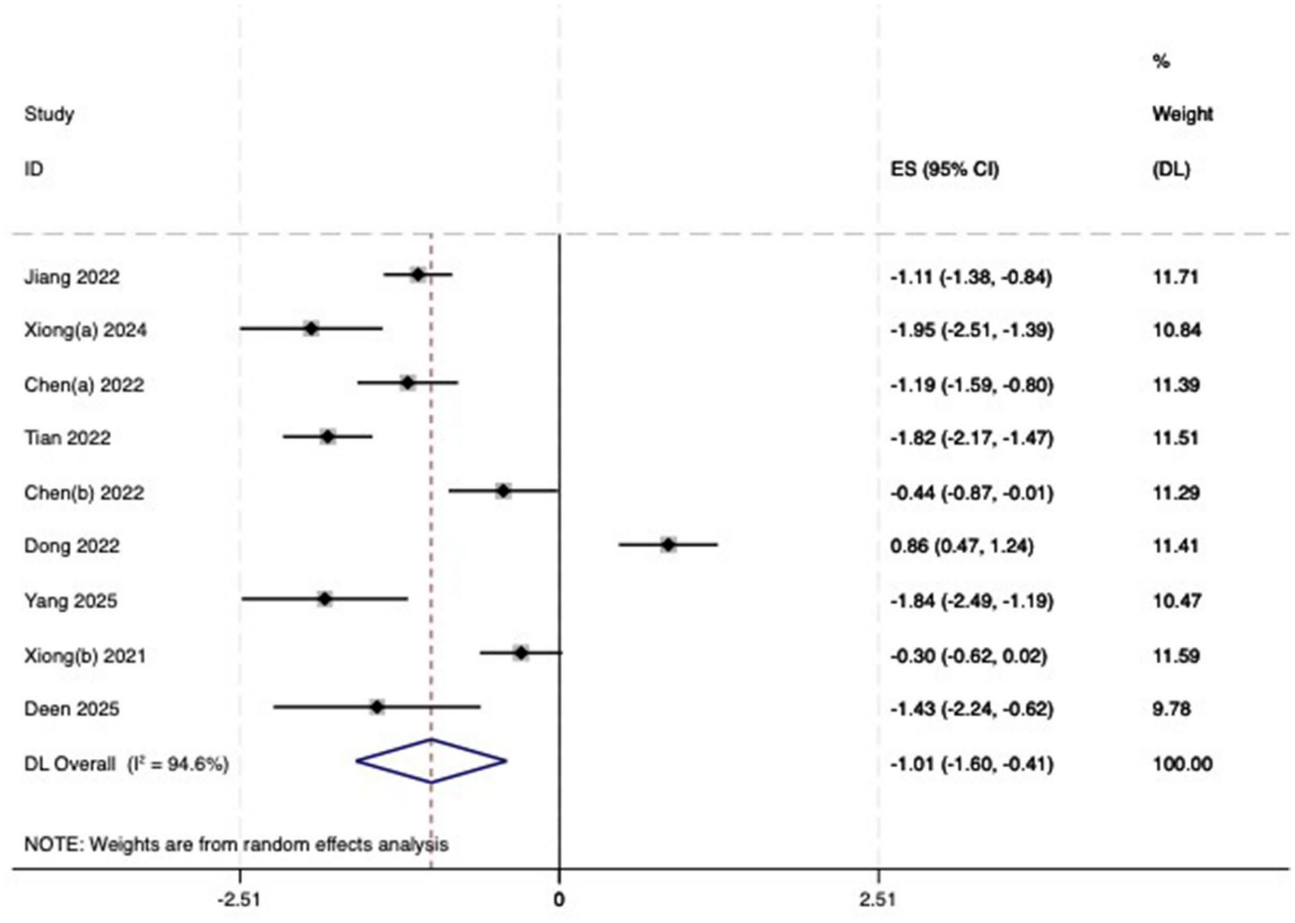
Forest plot of AL.

**Figure 8:**
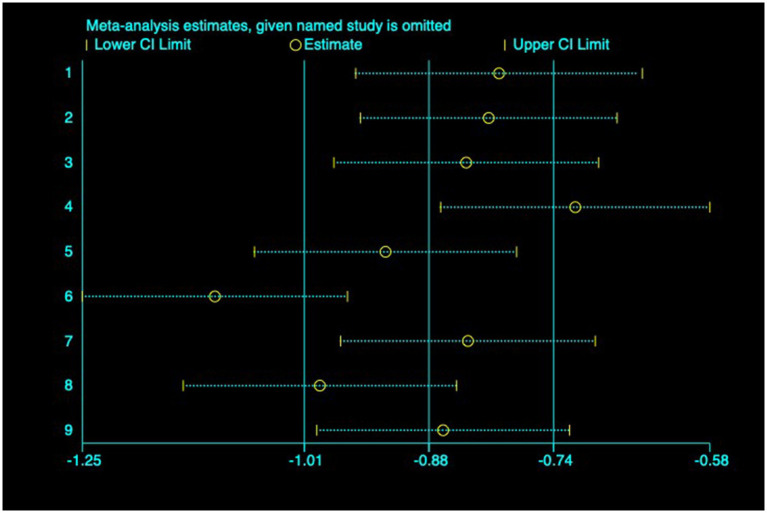
Sensitivity analysis of AL.

**Figure 9:**
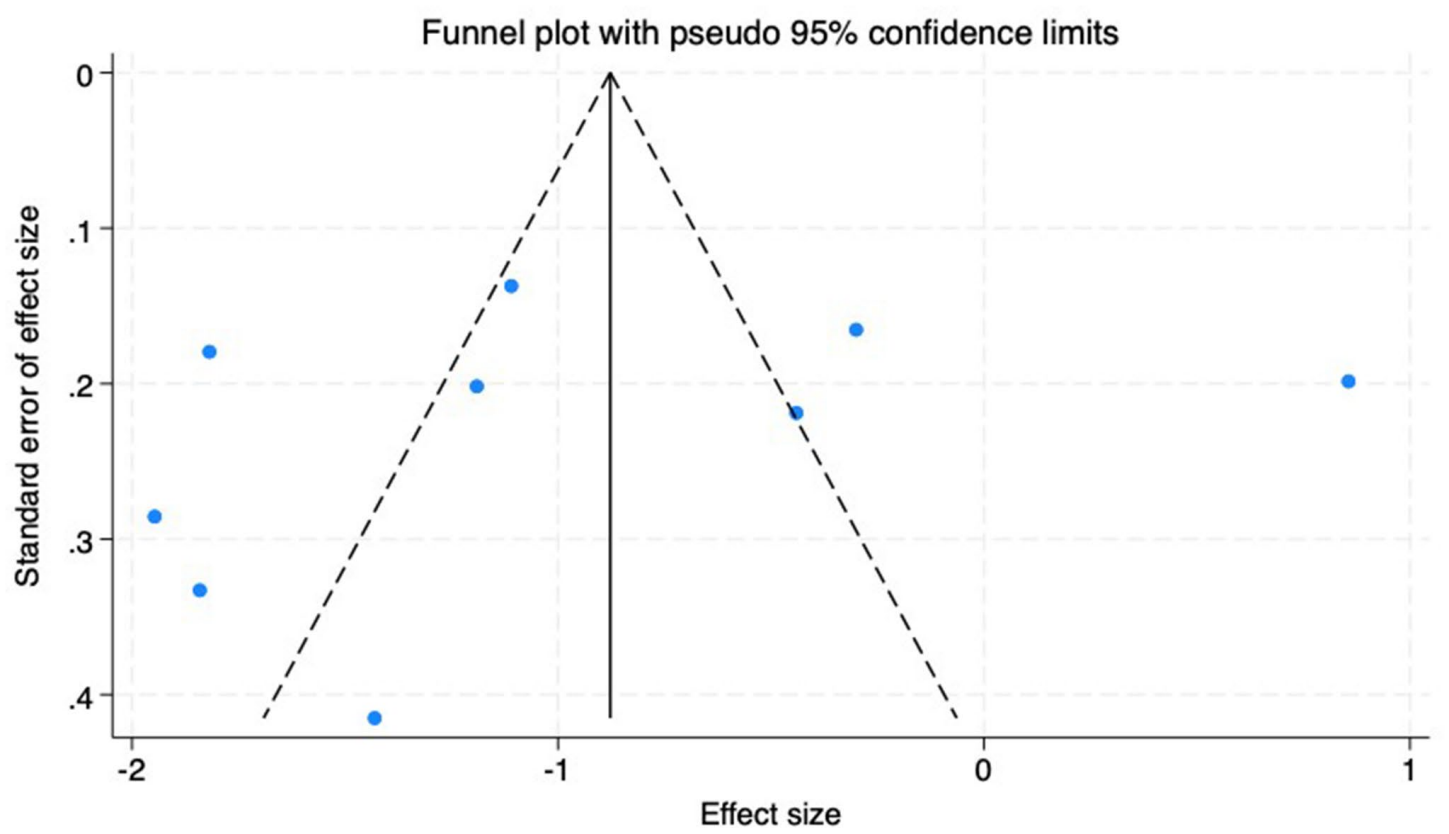
Funnel plot of the AL.

(1) SER: Nine studies (23–31) reported the baseline, median, or changes of SER before and after myopia intervention with RLRL, thus being included in the analysis. The combined results showed strong heterogeneity among the studies (*p* < 0.001, *I*^2^ = 85.2%), so the random-effects model was used to pool the effect sizes for analysis. The results indicate that the SER progressed more rapidly in the control group, while RLRL showed significant efficacy in reducing the increase in myopic SER, with the observed difference attaining statistical significance (ES = 0.84, 95% CI: 0.50–1.19, *p* = 0.004, Figure 4). Sensitivity analysis was performed by sequentially removing each included study. As shown in Figure 5, the results remained consistent with the original analysis, indicating that individual studies had minimal influence on the pooled results and indicating the robustness of the combined effect size. The funnel plot was employed to assess publication bias across nine research; the results indicate that the majority of points are situated within the 95% CI, exhibiting a degree of asymmetry. Further Egger’s test result is *p* = 0.816, suggesting no significant publication bias. As shown in Figure 6.

(2) AL: Among the included studies, nine studies (23–31) reported the AL before and after myopia intervention with RLRL. The difference in AL changes was computed before and after RLRL based on the endpoint and baseline data, and an analysis was conducted on it. The heterogeneity test indicated strong heterogeneity (*p* < 0.001, *I*^2^ = 94.6%). Consequently, the random effects model was selected to aggregate the effect sizes for analysis. The results showed that RLRL could effectively inhibit the increase in AL, and the difference was statistically significant (ES = −1.01, 95% CI: −1.60 to −0.41, *p* = 0.001, Figure 7). Sensitivity analysis was performed by sequentially removing each included study. As shown in Figure 8. Consistent with the original analysis results, single studies have minimal influence on the combined results, demonstrating that the combined effect value of this study is relatively stable. The funnel plot was used to detect publication bias in nine research, and it demonstrates that the majority of the points are distributed within the 95% CI, with some asymmetry. Further Egger’s test result is *p* = 0.5, suggesting no significant publication bias. As shown in Figure 9.

#### 3.3.2 Secondary outcome

**Figure 10:**
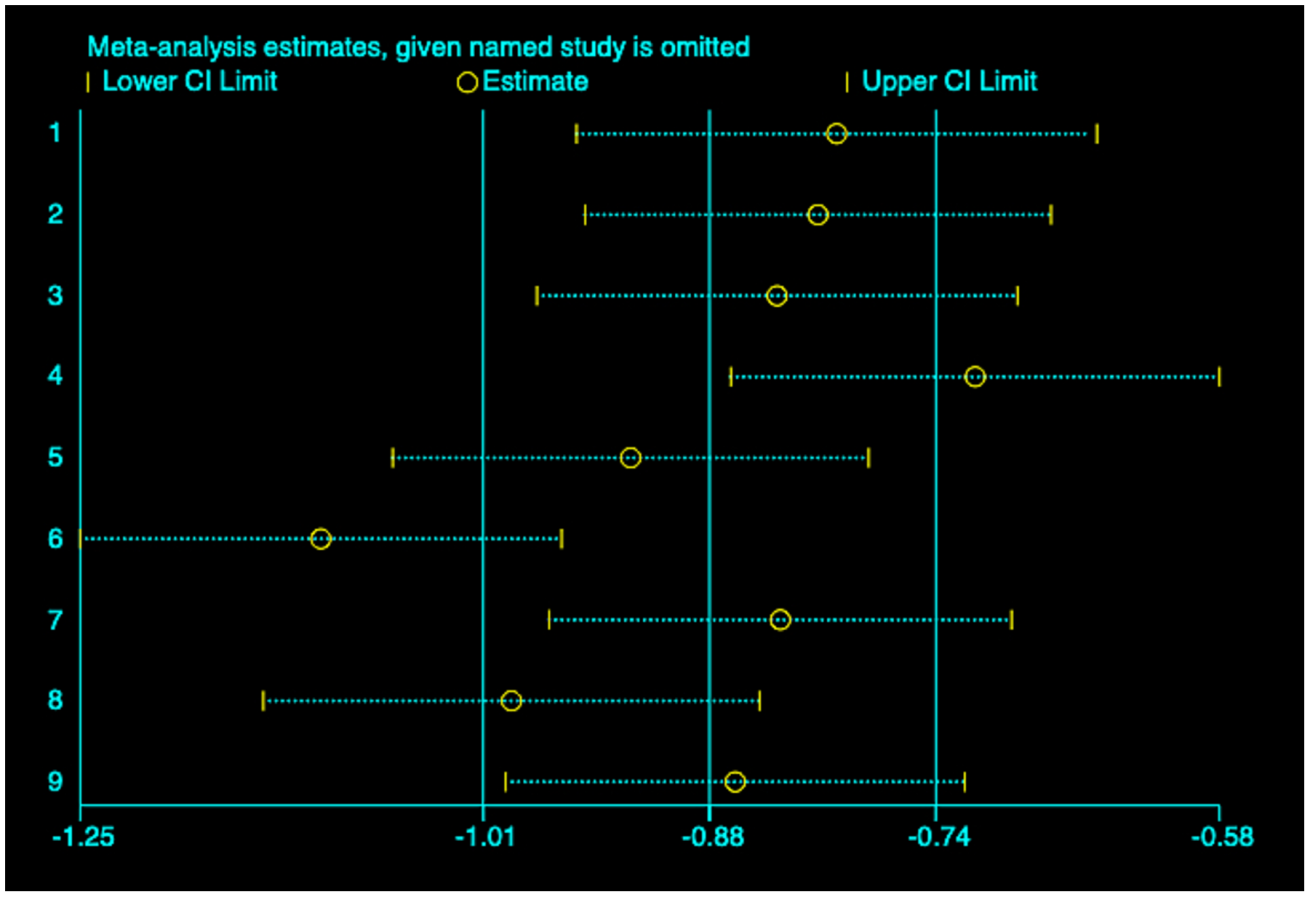
Forest plot of SFChT.

**Figure 11:**
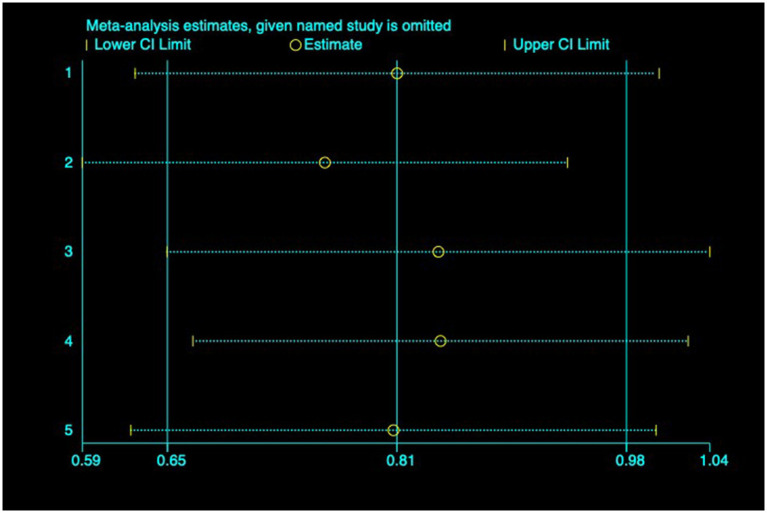
Sensitivity analysis of SFChT.

**Figure 12:**
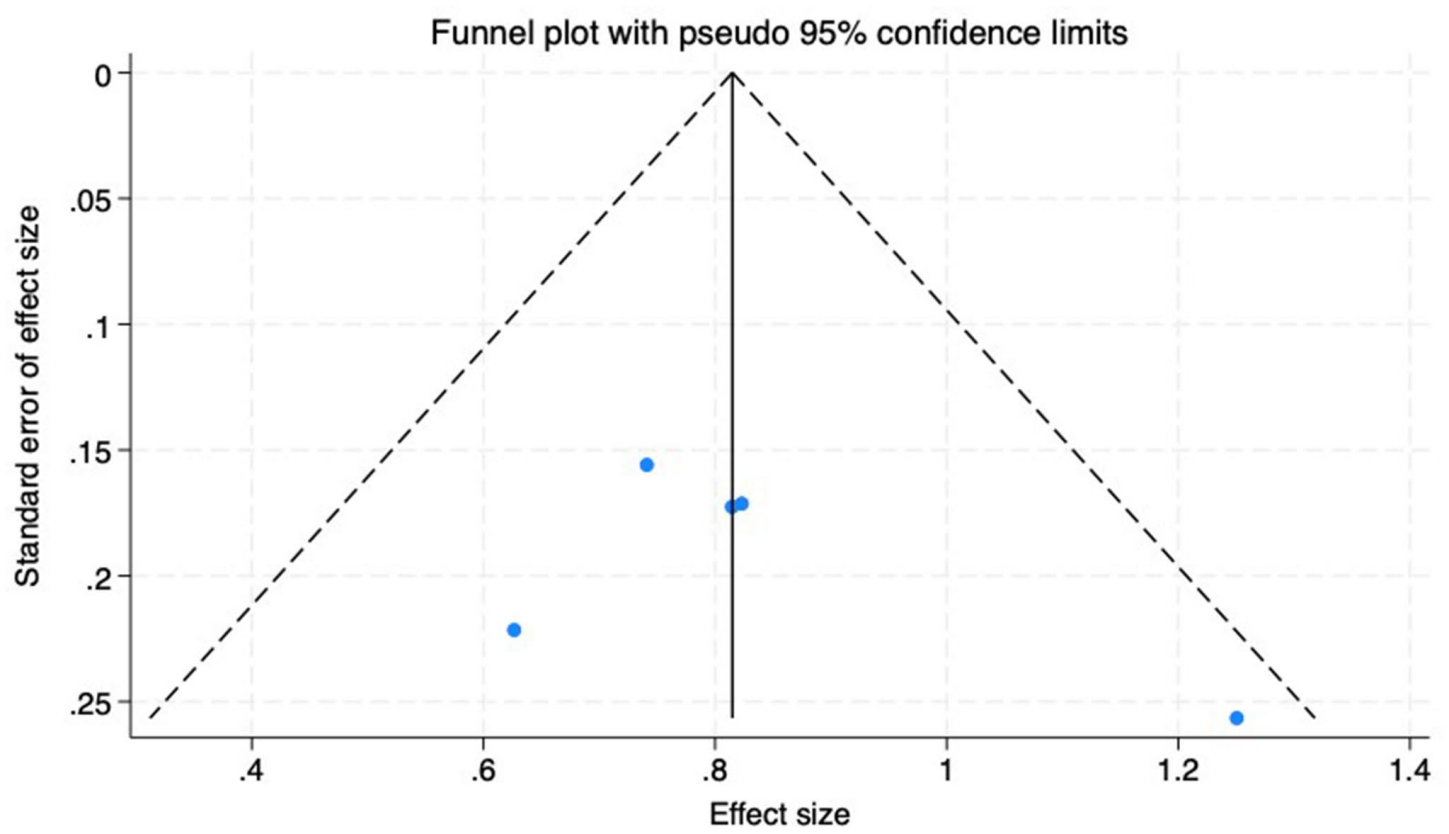
Funnel plot of the SFChT.

(3) SFChT: Among the included studies, five studies (23, 24, 26, 27, 30) reported the baseline median or change value of SFChT before and after myopia intervention with RLRL. In this study, relevant data were analyzed using a fixed-effect model to pool the effect sizes (*p* = 0.429, *I*^2^ = 0.0%). The meta-analysis results showed that the RLRL intervention could effectively increase SFChT, with statistical significance (SMD = 0.81, 95% CI: 0.65–0.98, *p* = 0.012, Figure 10). Sensitivity analysis was conducted on these studies, which showed reliable and stable results. The result is shown in Figure 11. We used the funnel plot to detect publication bias for five studies, and no sign of asymmetry was found. Also, Egger’s test did not suggest the existence of publication bias (*p* = 0.336), as shown in Figure 12.

#### 3.3.3 Subgroup analyses

The sources of heterogeneity were examined using techniques including regression analysis, subgroup analysis, and sensitivity analysis. A subgroup analysis was conducted based on classification criteria, including wavelength, output power, and irradiation scheme, but still failed to pinpoint the sources of heterogeneity. Therefore, the random effects model was selected, as shown in Supplementary Figures 1–8.

#### 3.3.4 GRADE evaluation for evidence quality

**Figure 13:**
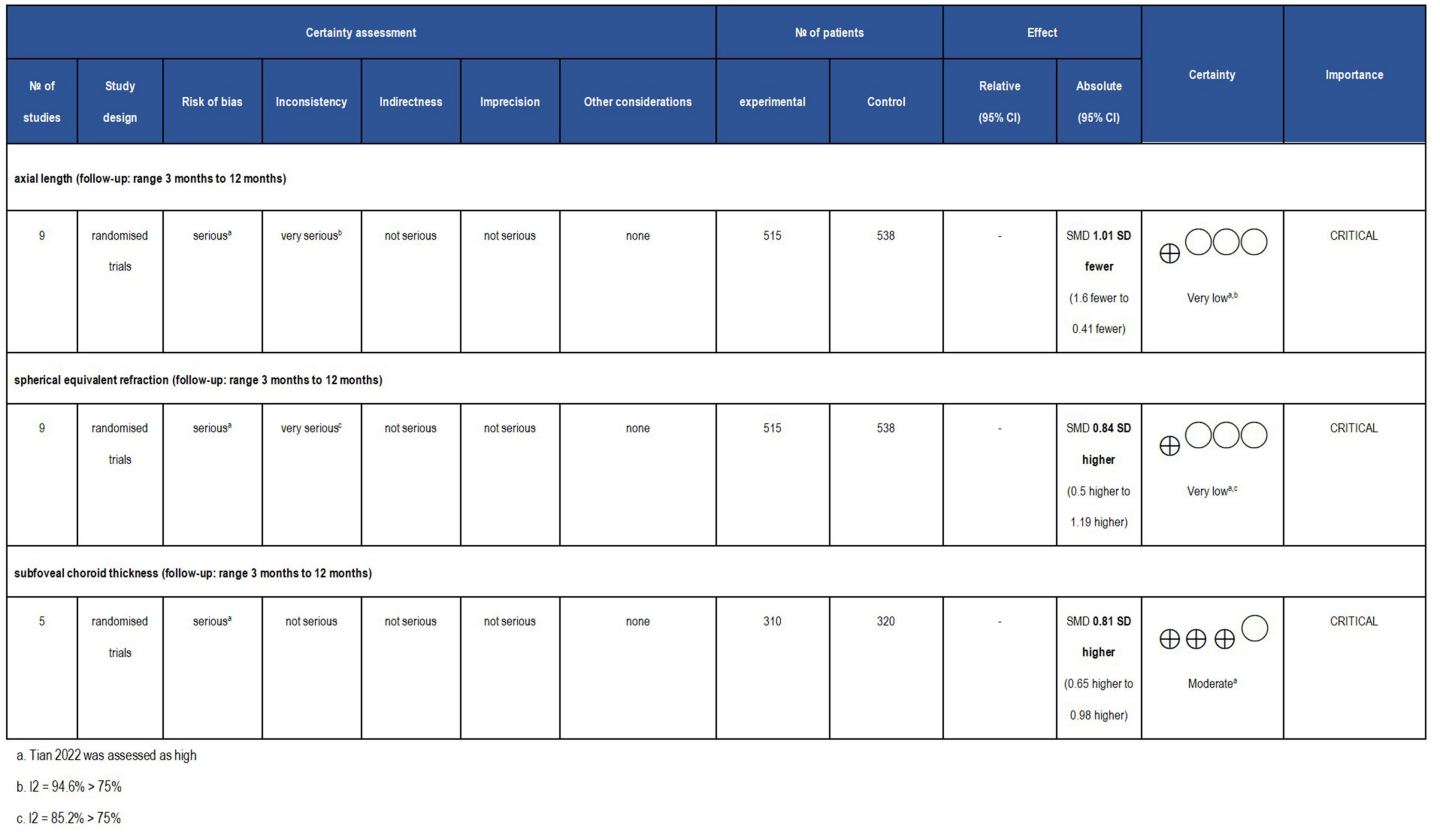
Grade analysis and certainty of evidence. The GRADE system clearly defines and evaluates evidence quality and recommendation levels, along with comprehensive standards for upgrading and degrading different evidence. The GRADE system classifies the quality of evidence as ‘high,’ ‘moderate,’ ‘low,’ and ‘very low’.

Although the included RCTs were represented as the evidence’s highest level, the quality of the outcomes should still be interpreted with caution. The outcome of SFChT was rated as moderate evidence due to risk of risk of bias concern. Moreover, the results of SER and AL were classified as very low-quality evidence due to the presence of bias and heterogeneity, as illustrated in Figure 13.

## 4 Discussion

This updated systematic review and meta-analysis concentrated on the effectiveness of RLRL therapy in children with myopia. In this meta-analysis involving nine RCTs with 1,053 participants, RLRL therapy reduced SER, shortened AL, and increased SFChT. It is important to highlight that acute choroidal thickening following photo biomodulation may not indicate enduring structural changes, and the clinical implications are still ambiguous. Although both primary and secondary outcomes showed certain improvements, the follow-up period was relatively short. Consequently, it was tentatively hypothesized that RLRL therapy may successfully reduce axial length and enhance choroidal thickness in children with spherical equivalent refraction of ≤ − 6.00D, consequently decelerating myopia progression.

The systematic review and meta-analysis assessed publication bias and found no significant evidence of bias present. However, when calculating the combined effect size, high heterogeneity was observed. The substantial variation may be attributable to varying inclusion criteria for SER among studies. For example, Jiang et al. (23) included participants with SER ranging from −1.00D to −5.00D and astigmatism ≤ 2.5D, while Xiong et al. (24) analyzed the more myopic eye in the range from −0.5D to −6.00D and astigmatism ≤ 3D; and Chen et al. (25) included participants with SER from −1.00D to −6.00D and astigmatism ≤ 1.5D. Additionally, there were differences in the treatment methods used by the control groups. In Chen et al.’s (27) trial, the control group was administered low-dose atropine, whereas in Dong et al.’s (28) study, the control group received a 10% dummy device in conjunction with single-vision spectacles. Moreover, differences in the age of children among the included studies may also contribute to the high heterogeneity. For example, Jiang et al. (23) studied children aged 8–13 years, Tian et al. (26) examined children aged from 6 to 12 years, and Xiong et al. (30) included children aged 7–15 years. In this study, the age range of included children is relatively wide. Moreover, discrepancies existed across the manufacturers, wavelengths, and wattage of red-light therapy devices utilized across several trials. For example, Dong et al. (28) did not report the wavelength, while Deen et al. (31) reported using a wavelength of 650 ± 10 nm, and Chen et al. (27) reported a wavelength of 635 nm. Besides, there were differences in the study population and ethnicity. For example, Jiang et al. (23) and Xiong et al. (24) exclusively included Chinese participants, whereas Deen et al. (31) included people of many ethnicities, including Caucasians and Asians.

The existing strategies for myopia control include low-dose atropine, orthokeratology, multifocal lenses, increased outside exposure, gas-permeable contact lenses, and multifocal glasses. However, current treatments may not be suitable for all patients with myopia, and new treatments are needed to slow its progression. Recent research indicates that low-level red-light therapy can slow myopia progression by significantly reducing changes in AL (32). Through meta-analysis, this study shows that RLRL therapy effectively slows the progression of childhood myopia. Notably, variations in device characteristics (wavelength, power, exposure time, and frequency) are expected to contribute significantly to clinical outcome heterogeneity.

Current studies suggest that the occurrence and progression of myopia may be affected by multiple factors, such as oxidative stress and inflammation (33). Oxidative stress causes harm to ocular tissues, including the retina and sclera, through the overproduction of reactive oxygen species (ROS), which can potentially lead to axial length growth (34–36). Red light therapy has been applied to retinal diseases (37, 38), including age-related macular degeneration, diabetic retinopathy, and hereditary optic nerve disorders. In recent years, some studies have suggested that red light irradiation may have multiple potential benefits for myopia. It promotes the synthesis of adenosine triphosphate by mitochondria, enhancing cellular energy levels, while concurrently reducing the production of ROS and alleviating the harmful effects of oxidative stress on retinal cells, thereby providing a protective role (39, 40). Secondly, red light can also improve the bioavailability of nitric oxide (41). Red light at 670 nm can interact with nitric oxide synthase, effectively reducing oxidative stress response (42). In addition, scleral tissue may be a key target of RLRL therapy (43). In instances of significant myopia, diminished oxygen availability to the retinal pigment epithelium and choroid intensifies oxidative stress, initiates cellular responses, and compromises the sclera by breaking down collagen, resulting in increased elasticity and susceptibility to elongation (44, 45). For instance, subjecting human scleral fibroblasts cultivated in a hypoxic environment to 660 nm light might enhance the synthesis of collagen 1a1 and downregulate hypoxia-inducible factor (HIF)-1α, therefore reducing axial development (46, 47). However, there are still many controversies regarding RLRL therapy. For example, in published clinical trials on RLRL therapy, significant differences are observed in the specified laser power, wavelength, and model. The power ranges from 0.16 to 2.0 mW, and the wavelength varies from 635 to 670 nm. Although some commercially available laser systems have met medical device regulations in Europe and the Asia-Pacific region, current studies have not determined the particular wavelengths and power settings that are most effective for suppressing axial growth. Therefore, it is necessary to determine safe exposure levels and strict manufacturing standards. Moreover, the cumulative damage of short-wavelength light to ocular tissues only becomes apparent after long-term exposure (48). Importantly, An increase in choroidal thickness mechanically displaces the retina anteriorly, hence reducing the AL. The choroid is rich in blood vessels, and variations in choroidal blood flow might influence the AL. As myopia progresses, choroidal thickness and blood flow decrease, leading to ischemia and hypoxia of the choroid; this, in turn, perpetuates a further decline in choroidal thickness, creating a detrimental cycle. The use of RLRL irradiation may help improve choroidal blood flow, thus reversing the vicious cycle mentioned above (49).

While no adverse effects were documented in the included randomized controlled trials, this does not imply that complications, such as vision loss, will not arise during prolonged treatment. Adverse effects, including skin irritation and ocular pain, may occur in certain instances while utilizing red light treatment for myopia. Additionally, the recurrence of myopia after treatment has not been thoroughly investigated and long-term retinal safety remains unknown. Consequently, it is crucial to evaluate the potential danger of delayed long-term detrimental consequences on retinal function and health resulting from extended red-light therapy. This will likewise serve as a focal point for our future research.

This study has several limitations. Firstly, 90% of the included studies were conducted in China, and only one study was conducted in Australia, which included a multi-ethnic population. Research evidence demonstrates that ocular parameters differ by ethnicity, with myopia progression in Chinese children occurring more rapidly than in Caucasian children (50, 51). This suggests that ethnic differences play a significant role in the progression of myopia and may impact the effectiveness of interventions. Secondly, there are few studies on RLRL therapy for controlling myopia progression. Although the RCT method provides compelling evidence, the analysis based on the summary of small-sample studies is unstable. Thirdly, the longest treatment duration in the included studies was 12 months. Despite the absence of reported side events during treatment, the long-term efficacy and safety of RLRL therapy, together with the regression and rebound effects post-discontinuation, necessitate additional investigation. Fourth, the results may have been influenced by the predominance of Chinese cohorts, brief follow-up durations, diverse control groups, and a significant risk of publication bias. However, this study used a random-effects model and sensitivity analyses to verify the reliability and robustness of the findings. Ultimately, several included RCTs exhibit a significant risk due to departures from targeted interventions and inadequate masking. This is also a limitation of this study.

## 5 Conclusion

The current investigation reveals a notable disparity in SER, AL, and SFChT between children in the RLRL groups and the control groups. Based on our analysis, the intervention suggests potential effectiveness in slowing myopia progression and delaying axial elongation in children. Nevertheless, most of the studies are Chinese studies in this meta-analysis, which carry a certain risk of bias. Upcoming research from multiple countries is expected to enhance the evidence base for this therapy method. Additionally, there is a compelling need for more rigorous randomized controlled trials with lengthy follow-up periods to verify the long-term efficacy and safety of RLRL treatment in the management of myopia. However, the majority of included research employed the same commercial device series, which may limit generalizability and create manufacturer biases.

## Data Availability

The datasets presented in this study can be found in online repositories. The names of the repository/repositories and accession number(s) can be found in the article/Supplementary material.
