# Supplementary figures and images for: Exploring the efficacy of repeated low-level red-light therapy in retarding childhood myopia progression: updated systematic review and meta-analysis
Source: Front Med (Lausanne). 2026 Jan 22;13:1713885. doi: 10.3389/fmed.2026.1713885 (PMC12872480; doi:10.3389/fmed.2026.1713885)

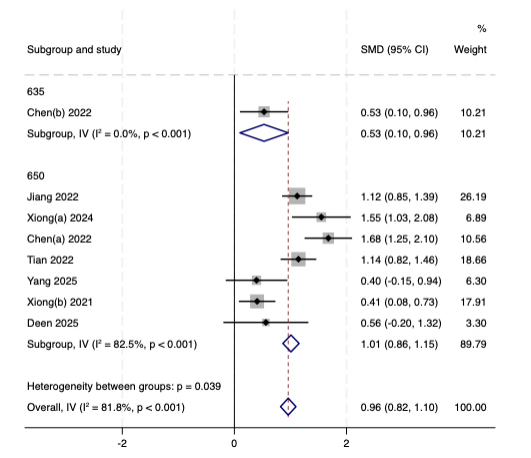

Supplement: Supplementary file 1 [file Image_1.TIF]

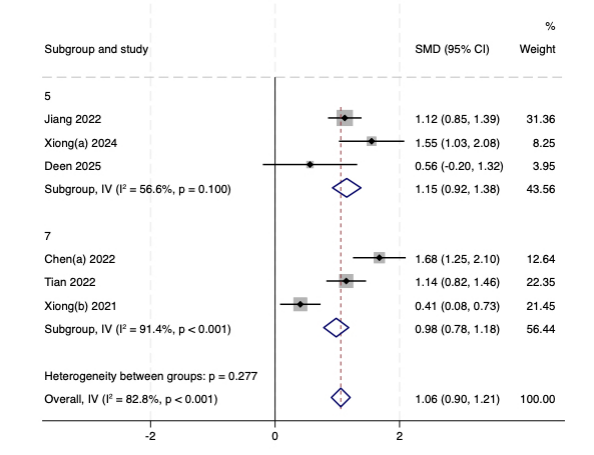

Supplement: Supplementary file 2 [file Image_2.TIF]

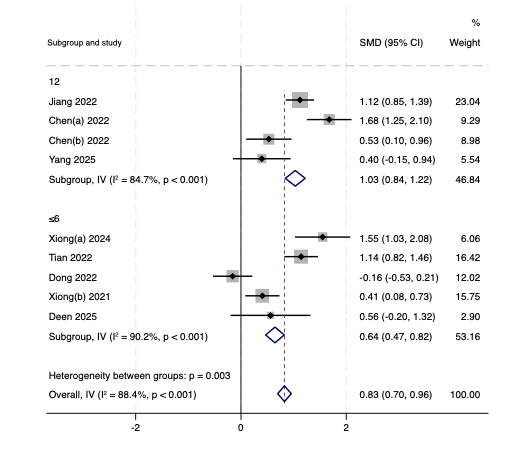

Supplement: Supplementary file 3 [file Image_3.TIF]

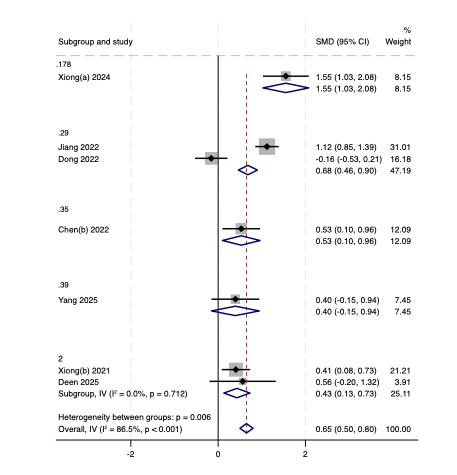

Supplement: Supplementary file 4 [file Image_4.TIF]

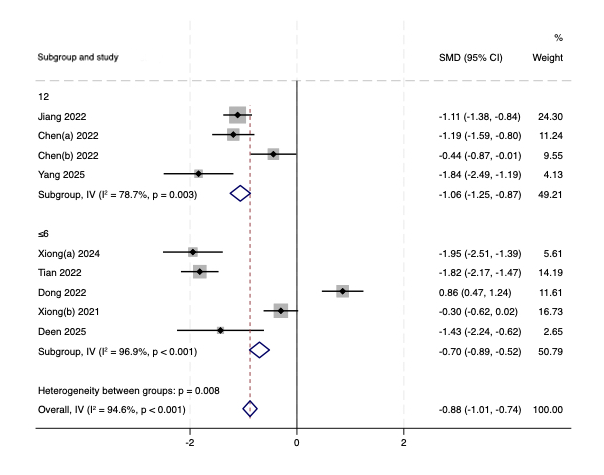

Supplement: Supplementary file 5 [file Image_5.TIF]

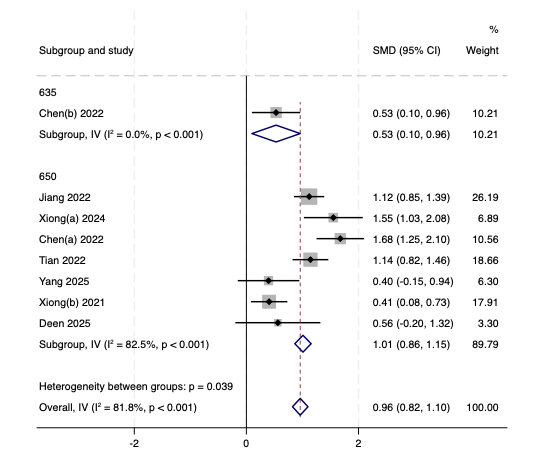

Supplement: Supplementary file 6 [file Image_6.TIF]

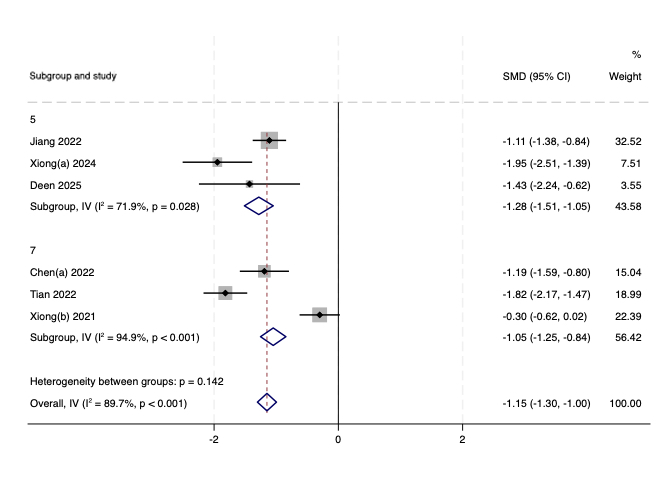

Supplement: Supplementary file 7 [file Image_7.TIF]

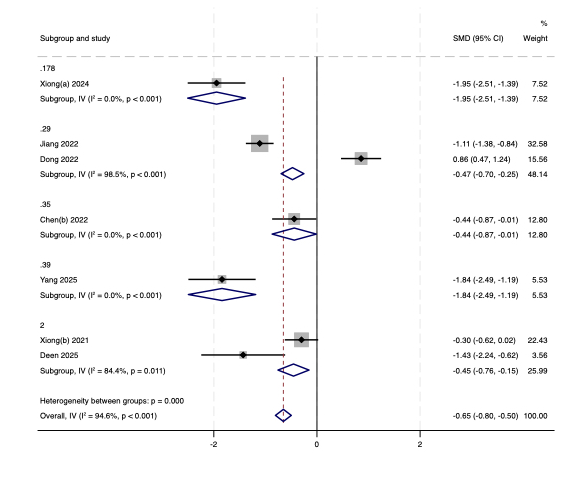

Supplement: Supplementary file 8 [file Image_8.TIF]
